# AWARENESS OF AGE-RELATED GAINS AND LOSSES AND THEIR ASSOCIATIONS WITH PHYSICAL AND COGNITIVE FUNCTION

**DOI:** 10.1093/geroni/igac059.2007

**Published:** 2022-12-20

**Authors:** Sarah Barber, Vanessa Martinez Lenis, Kate Hamel

**Affiliations:** Georgia State University, Atlanta, Georgia, United States; Georgia State University, Atlanta, Georgia, United States; San Francisco State University, San Francisco, California, United States

## Abstract

Awareness of age-related change (AARC) refers to people’s recognition of how aging has affected their performance, behavior, and ways of experiencing life. Sometimes these age-related changes are perceived as losses (AARC-Losses), such as when people notice declines in their health. However, other times these age-related changes are perceived as gains (AARC-Gains), such as when people notice they have developed a better sense of what is important to them. Past research has shown that higher AARC-Losses (and to a lesser extent lower AARC-Gains) are associated with poorer self-rated health. However, no research has yet examined whether AARC also relates to an objective performance-based measure of health. To address this, we examined the cross-sectional relationships between AARC-Losses and AARC-Gains with gait speed (i.e., a measure of physical function) in 164 community-dwelling older adults. Participants in this study also completed health-related questionnaires and the NIH Toolbox Cognition Battery. Results showed that AARC-Losses were most strongly predicted by depression levels, but higher AARC-Losses were also predicted by slower gait speeds. A different pattern emerged for AARC-Gains. After controlling for demographic factors, depression, and other self-reported measures of health, we found that higher AARC-Gains were predicted by poorer cognition and slower gait speed. The counterintuitive relationship between AARC-Gains and objective cognition has previously been reported in the literature. However, this study is the first to document that AARC-Gains are also associated with poorer performance on an objective measure of physical function. We discuss features of the AARC questionnaire that may lead to these paradoxical effects.

